# Improving OCTA Visualization of Macular Neovascularization via a Grayscale Inversion Method

**DOI:** 10.3390/life15101512

**Published:** 2025-09-25

**Authors:** Shinichiro Chujo, Yu-Chien Chung, Alberto Quarta, Hyunduck Kwak, Ceren Soylu, Rouzbeh Abbasgholizadeh, Mai Alhelaly, Raiyna Rattu, Giulia Corradetti, Muneeswar Gupta Nittala, Srinivas R. Sadda

**Affiliations:** 1 Doheny Image Reading and Research Lab, Doheny Eye Institute, 150 N. Orange Grove Blvd, Pasadena, CA 91103, USA; schujo@doheny.org (S.C.);; 2 Department of Ophthalmology, David Geffen School of Medicine, University of California Los Angeles, Los Angeles, CA 90095, USA; 3 Department of Ophthalmology, Graduate School of Medicine, Mie University, Tsu City 514-8507, Japan; 4 Department of Ophthalmology, Fu Jen Catholic University Hospital, Fu Jen Catholic University, New Taipei City 24205, Taiwan; 5 Department of Neurosciences, Imaging and Clinical Sciences, University “G. d’Annunzio” Chieti-Pescara, 66100 Chieti, Italy; 6 Ophthalmology Department, Tanta University, Tanta 31111, Egypt

**Keywords:** optical coherence tomography angiography, neovascular age-related macular degeneration, polypoidal choroidal vasculopathy, grayscale-inverted OCTA

## Abstract

Background: Age-related macular degeneration is a major cause of vision loss, and improved visualization of macular neovascularization (MNV) on OCT angiography (OCTA) could enhance clinical assessment. This study aimed to establish a simple and accessible image enhancement method. Methods: We retrospectively analyzed 24 eyes from 22 patients with MNV at the Doheny UCLA Eye Centers. Grayscale-inverted OCTA images were generated using the basic “Invert” function in ImageJ 1.51 23. Each original and inverted image pair was assessed for seven MNV-related features: structure and area within 3 × 3 mm, 6 × 6 mm and 12 × 12 mm scans, and presence of polypoidal lesions. Twenty-one ophthalmologists graded visibility using a standardized five-point scale. Paired comparisons were performed using the Wilcoxon signed-rank test. Results: Grayscale inversion significantly improved the visualization of MNV structure in 6 × 6 mm scans (mean difference: +0.67 ± 1.02; *p* = 0.008), 12 × 12 mm scans (+0.62 ± 1.07; *p* = 0.013), and detection of polypoidal lesions (+0.43 ± 0.98; *p* = 0.030). No significant differences were found for 3 × 3 mm structure (*p* = 0.793) or area-related features (all *p* > 0.3). Conclusions: Grayscale inversion may enhance MNV visibility and polypoidal lesion detection on OCTA. As this study relied solely on subjective assessments, future work should incorporate quantitative image analysis.

## 1. Introduction

Optical coherence tomography angiography (OCTA) is a minimally invasive imaging modality that allows for the visualization of macular neovascularization (MNV), and it has become widely used in the management of various macular diseases, including neovascular age-related macular degeneration (nAMD) [1,2]. OCTA produces vascular flow images by detecting changes in signal intensity across sequential B-scans obtained at the same retinal location, primarily capturing the movement of red blood cells, without the need for contrast dye [3]. However, OCTA has certain limitations. In lesions with blood flow below the detection threshold—such as polypoidal choroidal vasculopathy (PCV) or small MNVs—the flow signal may be weak and of insufficient contrast, making these lesions difficult to visualize [4,5,6,7]. To address these limitations, a method called variable interscan time analysis (VISTA) has been developed. VISTA captures images at multiple interscan times to quantify flow-related signal changes according to blood flow velocity [8,9]. Nevertheless, VISTA requires specialized imaging equipment, limiting its use in routine clinical settings and in many research environments.

In the field of internal medicine, the grayscale inversion technique—reversing black and white tones—has long been employed in radiographic imaging to enhance the visibility of vascular structures and lesions [10,11,12]. This approach is based on the principle of photographic positivity, whereby contrast is relatively amplified through tone inversion, thereby improving the human visual system’s ability to detect structural features [13]. In ophthalmology as well, black-on-white display modes have been reported for OCT images, suggesting that this visualization technique may also be applicable to ophthalmic imaging [14].

Based on this background, we hypothesized that applying the grayscale-inverted technique to OCTA images could enhance the visual identification and structural assessment of MNV lesions. If effective, this approach could serve as a readily implementable visualization method that does not require specialized equipment, thereby offering utility in both clinical and research settings. In this study, we evaluated whether grayscale-inverted OCTA images improve the visual assessment of MNV lesion location and morphology.

## 2. Materials and Methods

### 2.1. Study Design

This was a single-center, retrospective, observational, cross-sectional study conducted at the Doheny UCLA Eye Center Retina Clinics between January 2019 and April 2025. The study adhered to the tenets of the Declaration of Helsinki and was approved by Institutional Review Board and the Ethics Committee of the University of California—Los Angeles (approval number: IRB-15-0083).

### 2.2. Subjects

A total of 183 eyes of 161 patients diagnosed with neovascular age-related macular degeneration (nAMD) at the Doheny UCLA Eye Centers between January 2019 and April 2025 were included in this study. OCTA images (PLEX Elite 9000, Carl Zeiss AG, Jena, Germany) were obtained at baseline and at various postinjection time points. Macular cube scans with 3 × 3 mm, 6 × 6 mm, and 12 × 12 mm scan patterns were used to evaluate MNV, ensuring that the entire MNV structure was captured. Not all subjects underwent all three scan patterns, as acquisition was based on routine clinical care rather than a standardized study protocol. For each scan size, images were independently selected from the available scans that were of sufficient quality and clearly depicted the MNV. Selection was not influenced by patient identity or lesion characteristics. The MNV was further assessed using the built-in MNV analysis mode of the OCTA device. Image clarity was optimized by adjusting the slab position from the outer nuclear layer to either the retinal pigment epithelium (RPE) or Bruch’s membrane. From these images, those in which the MNV was clearly delineated and suitable for evaluation were selected by two experienced ophthalmologists (SC, YCC), who confirmed the image quality and accuracy.

### 2.3. Image Selections and Evaluation Criteria

Grayscale-inverted images were generated using the basic “Invert” function in ImageJ, which reverses the pixel intensity by subtracting each original grayscale value from 255. This operation transforms dark areas into light areas and vice versa, enhancing contrast without altering spatial resolution (Supplementary Video S1).

**Figure 1:**
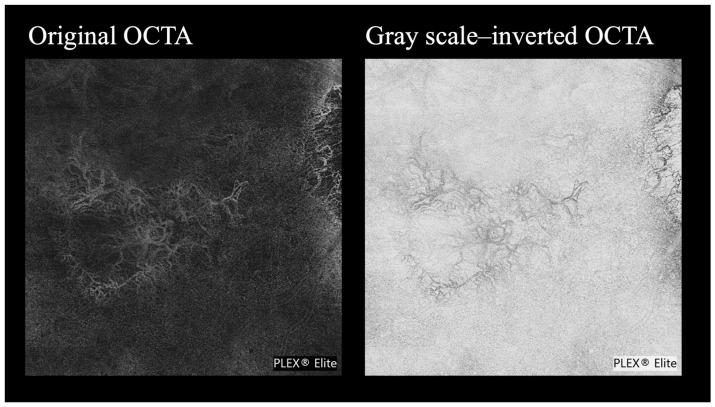
Comparison of original and grayscale-inverted OCTA images. The **left** panel shows the original OCTA image, and the **right** panel shows the corresponding grayscale-inverted OCTA generated using the “Invert” function in ImageJ.

The evaluation of image visibility was performed through subjective assessments by graders, comparing the original OCTA images (conventional OCTA) with the corresponding grayscale-inverted OCTA images created by applying grayscale inversion (Figure 1).

To evaluate for differences in lesion size and scan field, the evaluation was conducted on images acquired with 3 mm, 6 mm, and 12 mm scan patterns. For each scan size, two aspects of the MNV lesion were assessed: structure and area. In addition, the ability to detect polypoidal lesions—previously reported as difficult to identify using OCTA—was also evaluated.

The evaluation items were defined by the assessors as follows:

- **Structure**: how clearly can the overall vascular structure of the MNV, including peripheral branches, be visualized?
- **Area**: how clearly can the entire extent and margins of the MNV be understood?
- **Polypoidal lesions**: how clearly can the lesion’s shape, margins, and any branching features be understood?

**Figure 2:**
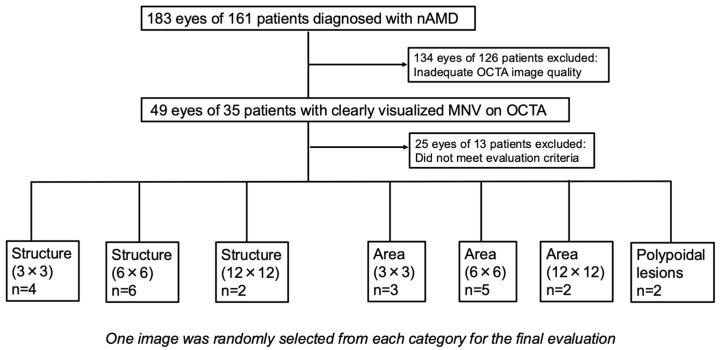
Flowchart of image selection for evaluation. Images were selected by two independent ophthalmologists who did not participate as raters in the subjective evaluation. Random selection was performed using the RAND function in Excel 16.100. nAMD, neovascular age-related macular degeneration; MNV, macular neovascularization; OCTA, optical coherence tomography angiography.

After categorizing the images according to each evaluation item, two independent ophthalmologists (SC and YCC) performed the categorization. A total of 49 eyes from 35 patients were initially included. Of these, 24 eyes from 22 patients met the evaluation criteria and were ultimately used for the final analysis. Finally, one image per evaluation item was randomly selected from these eyes using the RAND function in Excel (Figure 2).

And the raters who performed the final evaluation consisted of ophthalmologists who had completed the pilot evaluation and demonstrated an understanding of the evaluation criteria and study objectives. To prevent any learning effect, images used in the pilot phase were excluded from the final assessment. The two ophthalmologists (SC and YCC) who had selected the evaluation images were excluded from the pool of raters.

The following questions were used for the subjective evaluation and were applied uniformly in both the pilot and final assessments:

- **For structure**: which image provides a clearer visualization of the branching pattern and overall morphology of the MNV?
- **For area**: which image provides better visualization of the MNV boundaries and lesion area?
- **For polypoidal lesions**: which image provides a clearer visualization of the structure of the polypoidal lesion?

**Figure 3:**
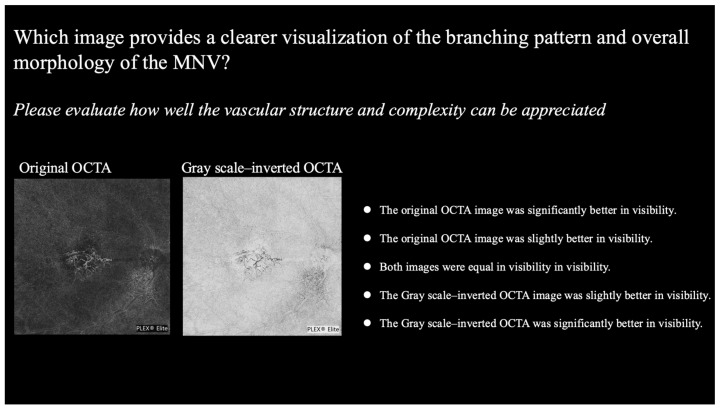
Comparison panels for subjective assessment of original vs. inverted OCTA images. Representative image pair used for the evaluation of vascular structure in 12 × 12 mm scans. Each rater graded the visibility on a five-point scale ranging from −2 (favoring the original OCTA image) to +2 (favoring the grayscale-inverted image), and these scores were subjected to statistical analysis. OCTA, optical coherence tomography angiography.

Each image pair was rated using a five-point scale as follows: −2, original OCTA image was significantly better; −1, original OCTA image was slightly better; 0, both images were equal; +1, grayscale-inverted OCTA image was slightly better; and +2, grayscale-inverted OCTA was significantly better (Figure 3). All images used for each evaluation criterion are provided in Supplementary Figure S1.

### 2.4. Pilot Evaluation

**Figure 4:**
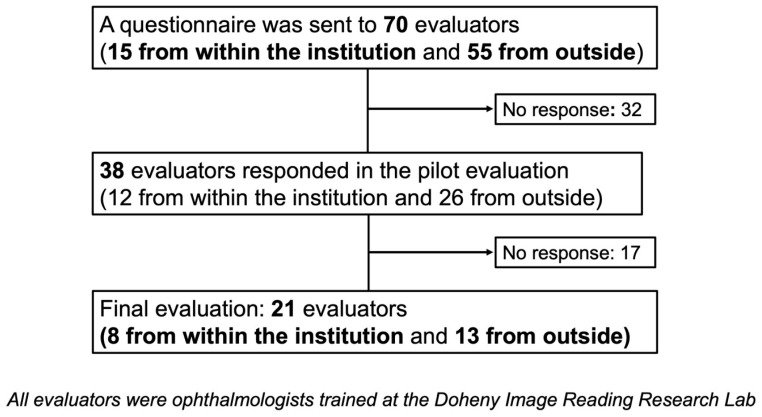
Flowchart of rater selection process. A total of 70 ophthalmologists were invited to participate in the pilot evaluation, of whom 38 completed it. Among these, 21 ophthalmologists responded to the main evaluation and were included as final raters.

Initially, the pilot evaluation questionnaire was distributed to 70 ophthalmologists (15 from within the Doheny Image Reading and Research Lab (DIRRL) and 55 from outside institutions, all of whom had been trained in the DIRRL in the past). A total of 38 ophthalmologists (12 from within the institution and 26 from outside) responded to the pilot evaluation (Figure 4).

### 2.5. Final Evaluation

A total of 38 ophthalmologists who completed the pilot evaluation were subsequently invited to participate in the final assessment, and responses were obtained from 21 ophthalmologists (8 from within the institution and 13 from outside), who were designated as the final raters (Figure 4).

### 2.6. Statistical Analyses

Responses were classified as positive responses of +1 or +2 favoring grayscale-inverted OCTA, or negative response of −1 and −2 favoring original OCTA, or neutral response or no preference of 0. Under the hypothesis that grayscale-inverted images would provide superior visibility compared to original OCTA images, a Wilcoxon signed-rank test was performed for each evaluation item, with *p* < 0.05 considered statistically significant. Additionally, the mean signed rank percentage (%) was calculated to quantify the relative superiority (or inferiority) of grayscale-inverted OCTA over the original OCTA for the different types of disorders. This percentage was derived from the ratio of signed rank sum to the total number of non-zero responses. In addition to the primary analysis using Wilcoxon signed-rank tests, inter-grader variability was further assessed by analyzing the distribution of grader responses for each evaluation item. The proportions of positive responses (+1 or +2), negative responses (−1 or −2), and neutral responses (0) were calculated to describe the consistency of grader preferences across evaluation items. These proportions were reported in the results section to supplement the primary analysis. All statistical analyses were performed using R version 2.9.0.

## 3. Results

The distribution of scores for each evaluation item was summarized separately for grayscale-inverted and original OCTA images (Table 1).

The mean ± standard deviation (SD) of score differences (grayscale-inverted image minus original image), along with corresponding *p*-values from Wilcoxon signed-rank tests, are summarized below. Significant differences favoring grayscale-inverted images were observed for MNV structure evaluations for both 6 × 6 mm (+0.67 ± 1.02; *p* = 0.008) and 12 × 12 mm (+0.62 ± 1.07; *p* = 0.013) scan sizes, as well as for polyp evaluations (+0.43 ± 0.98; *p* = 0.030). In contrast, no significant differences were found for 3 × 3 mm images for MNV structure (−0.19 ± 1.25; *p* = 0.793) or for any MNV area evaluations (3 × 3 mm area: −0.33 ± 1.11; *p* = 0.895; 6 × 6 mm area: +0.10 ± 1.26; *p* = 0.328; 12 × 12 mm area: −0.33 ± 0.66; *p* = 0.986). Inter-grader variability was further assessed by analyzing the proportions of positive (+1 or +2), negative (−1 or −2), and neutral (0) responses for each evaluation item (Table 2).

MNV structure evaluations for both 6 × 6 mm and 12 × 12 mm scans showed high positive response rates (61.9%) and relatively low neutral response rates (28.6% and 23.8%, respectively), indicating good grader consensus in favor of grayscale-inverted images. In contrast, area evaluations, particularly for the 12 × 12 mm scan (neutral: 61.9%), exhibited greater variability in grader responses.

Based on initial observations suggesting that original OCTA might outperform grayscale-inverted images in some MNV area evaluations, we conducted an additional analysis under the reverse hypothesis, assuming original OCTA to be superior for area assessment. This analysis revealed a significant difference in 12 × 12 mm scans (*p* < 0.05), suggesting that grayscale inversion may not improve visibility in all situations (Table 3).

## 4. Discussion

In this study, the basic Invert function in ImageJ was utilized to generate grayscale-inverted OCTA images, and their utility was compared with that of the original OCTA images for the evaluation of MNV in terms of structure, lesion area, and polypoidal lesion detection.

In the evaluation of MNV structure, grayscale-inverted OCTA images demonstrated significantly superior visibility compared with original images for both the 6 × 6 mm and 12 × 12 mm OCTA scans (Supplementary Figure S1B,C). These results suggest that grayscale inversion may be particularly useful for structural assessment when a wider scan field is used or when MNV lesions are small. It has been reported that as the scan field increases, the visualization of fine neovascular structures becomes more challenging [14]. According to Li et al., when comparing vascular structure parameters such as vessel density (VD) and vessel length density (VLD) between 3 × 3 mm and 6 × 6 mm scans using the Plex Elite system, the 3 × 3 mm images provided higher resolution and were more suitable for evaluating fine vascular structures, yielding higher VD and VLD values [14]. Furthermore, it has also been reported that as MNV lesions become smaller, the blood flow signal decreases, making it more difficult to visualize vascular structures [6,7]. This finding suggests that the effectiveness of grayscale inversion does not stem from enhancing the actual flow signal captured by OCTA, but rather from increasing the relative luminance contrast within the image. This, in turn, enhances the perceived brightness of the vascular structures and may function as a “booster effect” that improves their visual detectability. In contrast, no significant difference was observed between grayscale-inverted and original images in the 3 × 3 mm OCTA scans (Supplementary Figure S1A), This may be attributed to the inherently higher resolution of 3 × 3 mm OCTA images compared to the 6 × 6 mm or 12 × 12 mm scans, potentially diminishing the relative advantage of grayscale inversion [14]. These findings suggest that the use of inversion may be particularly beneficial for evaluating MNV structure in wider field OCTA images (≥6 mm) or in cases where the MNV lesion itself is small, regardless of the scan size.

Structural evaluation of MNV is an important aspect in clinical practice, and several studies have investigated the relationship between anti-VEGF treatment effects and structural parameters of MNV [15,16,17,18,19]. Takeuchi et al. reported that during the loading phase of anti-VEGF therapy, vascular density initially decreases but subsequently returns to baseline levels [18]. Additionally, Choi et al. demonstrated that deterioration in MNV branching complexity can occur prior to the recurrence of exudative features [19]. These findings highlight the clinical significance of accurately evaluating MNV structure as a means of assessing treatment response and suggest that grayscale inversion may serve as a useful tool to enhance visualization in this context.

Moreover, if grayscale inversion proves to be effective in visualizing small MNV lesions, it may also be applicable to the evaluation of myopic choroidal neovascularization (myopic CNV). Although the utility of OCTA in myopic CNV has been reported, the small size and weak flow signals of such lesions present challenges for accurate assessment [20,21]. In this regard, it is possible that grayscale inversion may offer benefits by improving lesion detectability in these cases as well. In this study, grayscale-inverted OCTA also appeared to be effective for the identification of polypoidal lesions (Supplementary Figure S1G). This effect is likely due to the enhancement of relative luminance contrast between the lesion and surrounding tissue, achieved through image inversion, thereby improving human visual detectability. Accurate detection and evaluation of polypoidal lesions are critically important in the diagnosis and management of polypoidal choroidal vasculopathy (PCV), as supported by multiple prior reports [22,23,24,25,26]. However, OCTA has known limitations in this context, as the slow blood flow within polyps that often results in weak flow signals, making lesion identification challenging. If grayscale inversion improves the visualization and structural evaluation of polypoidal lesions, it may not only enhance diagnostic accuracy, but also contribute to lesion quantification—such as polyp counts—and thus treatment response assessment in clinical practice.

In contrast, grayscale-inverted OCTA did not demonstrate utility in the assessment of lesion area, and intriguingly, additional analysis revealed that the original images were superior for MNV area assessment when using 12 × 12 mm OCTA scans (Supplementary Figure S1D–F). Several factors may account for this finding. First, delineating lesion boundaries is a relatively coarse evaluation compared to assessing the fine structure of MNV and may depend more heavily on the observer’s clinical experience as an ophthalmologist. This could have minimized differences in evaluation between the grayscale-inverted and original images. Second, given that MNV lesions were already well visualized in the original OCTA images acquired with the Plex Elite system (based on our selection process), the baseline visibility may have been sufficiently high that the grayscale inversion provided no additional benefit in terms of visual enhancement. These findings suggest that while the grayscale inversion method may enhance the visibility of MNV structure and polypoidal lesions, it may be less suitable for macroscopic assessments, such as lesion area or shape. Therefore, selecting between grayscale-inverted and original OCTA images based on the specific purpose of observation may represent a practical and effective strategy in both clinical and research settings.

The primary advantage of the grayscale inversion method presented in this study lies in its simplicity and high reproducibility. This technique only utilizes the basic “Invert” function included in ImageJ, an open-source software provided by the National Institutes of Health (NIH), and does not require any specialized macros or additional programming. As such, the method can be performed by anyone immediately and at no cost by simply installing ImageJ. Various image processing techniques aimed at enhancing MNV visibility on OCTA images have been reported previously; however, many of these approaches rely on artificial intelligence (AI)-based methods or advanced image processing algorithms [8,9,27,28]. While these advanced image processing techniques offer high precision and analytical capabilities, they often require specialized knowledge and computational resources, limiting their widespread adoption in routine clinical practice. In contrast, the grayscale inversion method employed in this study demonstrated improved visibility of MNV structures and polypoidal lesions through a remarkably simple operation. This highlights the clinical practicality of the method, and if its utility is further validated, it may be implemented as a standard function in OCTA devices and widely adopted in everyday clinical settings.

## 5. Limitations

There are several limitations in this study which must be considered when assessing the significance of our results. This study is based on visual subjective assessment, and we believe that further quantitative evaluation is essential to scientifically establish the utility of images. Based on the suggested superiority of inverted images in terms of structural features demonstrated in this study, a quantitative analysis of MNV structure—such as vessel length, junction density, and vessel diameter—has been initiated using NIH freeware tools such as AngioTool [16,29]. In addition, to objectively verify the utility of the images, a study evaluating intra- and inter-grader reliability has also been initiated. These quantitative investigations are expected to play an important role in supporting the usefulness of grayscale visualization, and the present study serves as a fundamental basis for them. And it is necessary to investigate whether grayscale-inverted OCTA images, compared to conventional OCTA images, can offer clinical impact in the assessment of anti-VEGF treatment efficacy—for example, by enhancing the visualization of MNV suppression before and after treatment—as well as in improving the accuracy of polypoidal lesion counting, which may influence the evaluation of therapeutic outcomes in PCV.

Ideally, to eliminate potential evaluation bias, a masked assessment between original and grayscale-inverted images would have been desirable in this study. However, because grayscale inversion introduces a distinct visual characteristic (i.e., black-and-white reversal), evaluators were able to recognize which images were inverted, making complete masking infeasible. Blinding was not feasible due to the nature of the study design. Nevertheless, since some evaluation items were rated higher in the original images, it is unlikely that there was a consistent positive bias favoring grayscale-inverted images. Furthermore, since only images in which MNV was deemed sufficiently well-visualized were included in the evaluation, the applicability of grayscale inversion to cases with poorly visualized MNV remains unclear and should be investigated in our ongoing reproducibility study. To more rigorously assess the consistency of subjective evaluations in this study, Inter-grader agreement analysis using the κ (kappa) statistic, along with appropriate multiple comparison correction, is planned for future studies.

Another limitation of this study is the selection of images with good visualization of the MNV lesion on the original OCTA images. It is possible that the benefit of grayscale inversion could be greater (or worse) in cases with somewhat reduced imaging quality. This will also need to be evaluated in future studies. To further investigate this issue, future studies may consider increasing the number of evaluators and OCTA images to strengthen the generalizability of the conclusions. If the sample size is increased in future studies, more generalized conclusions may be drawn, including potential associations between patient background characteristics—such as age, sex, hypertension, and other systemic factors—and MNV structural features. Additionally, comparative studies between experienced ophthalmologists and residents could help determine the educational value of this method.

## 6. Conclusions

This study demonstrated that applying a grayscale inversion technique—achieved by inverting OCTA images—may be useful for enhancing the visibility of MNV structure and identifying polypoidal lesions. On the other hand, for the evaluation of MNV lesion area, the original images appeared to be more suitable. These findings suggest that, in routine clinical practice using OCTA, clinicians may benefit from selectively utilizing either grayscale-inverted or original images depending on the specific diagnostic objective.

## Figures and Tables

**Table 1:** Distribution of subjective scores comparing inverted and original OCTA images across evaluation factors.

Evaluation Factor	Inverted >> Original (+2)	Inverted > Original (+1)	Inverted = Original (0)	Inverted < Original (−1)	Inverted << Original (−2)	*p*-Value	Mean ± SD
**Structure (3 × 3)**	0	10	1	5	5	0.793	−0.19 ± 1.25
**Structure (6 × 6)**	4	9	6	1	1	<0.01	0.67 ± 1.02
**Structure (12 × 12)**	4	9	5	2	1	<0.05	0.62 ± 1.07
**Area (3 × 3)**	0	3	9	6	3	0.895	−0.33 ± 1.11
**Area (6 × 6)**	4	4	4	8	1	0.328	0.60 ± 1.26
**Area (12 × 12)**	0	1	13	6	1	0.986	−0.33 ± 0.66
**Polypoidal lesions**	3	7	7	4	0	<0.05	0.43 ± 0.98

Inverted, grayscale-inverted OCTA; original, original OCT.

**Table 2:** Proportions of positive, negative, and neutral responses for evaluation factors.

Evaluation Factor	Positive Rate (%)	Negative Rate (%)	Neutral Rate (%)
**Structure (3 × 3)**	47.6	47.6	4.8
**Structure (6 × 6)**	61.9	9.5	28.6
**Structure (12 × 12)**	61.9	14.3	23.8
**Area (3 × 3)**	14.3	42.9	42.9
**Area (6 × 6)**	38.1	42.9	19.0
**Area (12 × 12)**	4.8	33.3	61.9
**Polypoidal lesions**	47.6	19.0	33.3

**Table 3:** Exploratory analysis assuming original OCTA is superior in lesion area evaluation.

Evaluation Factor	Inverted >> Original (+2)	Inverted > Original (+1)	Inverted = Original (0)	Inverted < Original (−1)	Inverted << Original (−2)	*p*-Value
**Area (3 × 3)**	0	3	9	6	3	0.121
**Area (6 × 6)**	4	4	4	8	1	0.69
**Area (12 × 12)**	0	1	13	6	1	<0.05

## Data Availability

The original contributions presented in this study are included in the article/Supplementary Material. Further inquiries can be directed to the corresponding author.

## Supplementary Materials

The following supporting information can be downloaded at: https://www.mdpi.com/article/10.3390/life15101512/s1, Figure S1: This figure includes all the images used for evaluation in the present study; Video S1: This video demonstrates the process of generating grayscale-inverted OCTA images using the Invert function in ImageJ.
