# Supplementary figures and images for: Improving OCTA Visualization of Macular Neovascularization via a Grayscale Inversion Method
Source: Life (Basel). 2025 Sep 25;15(10):1512. doi: 10.3390/life15101512 (PMC12565100; doi:10.3390/life15101512)

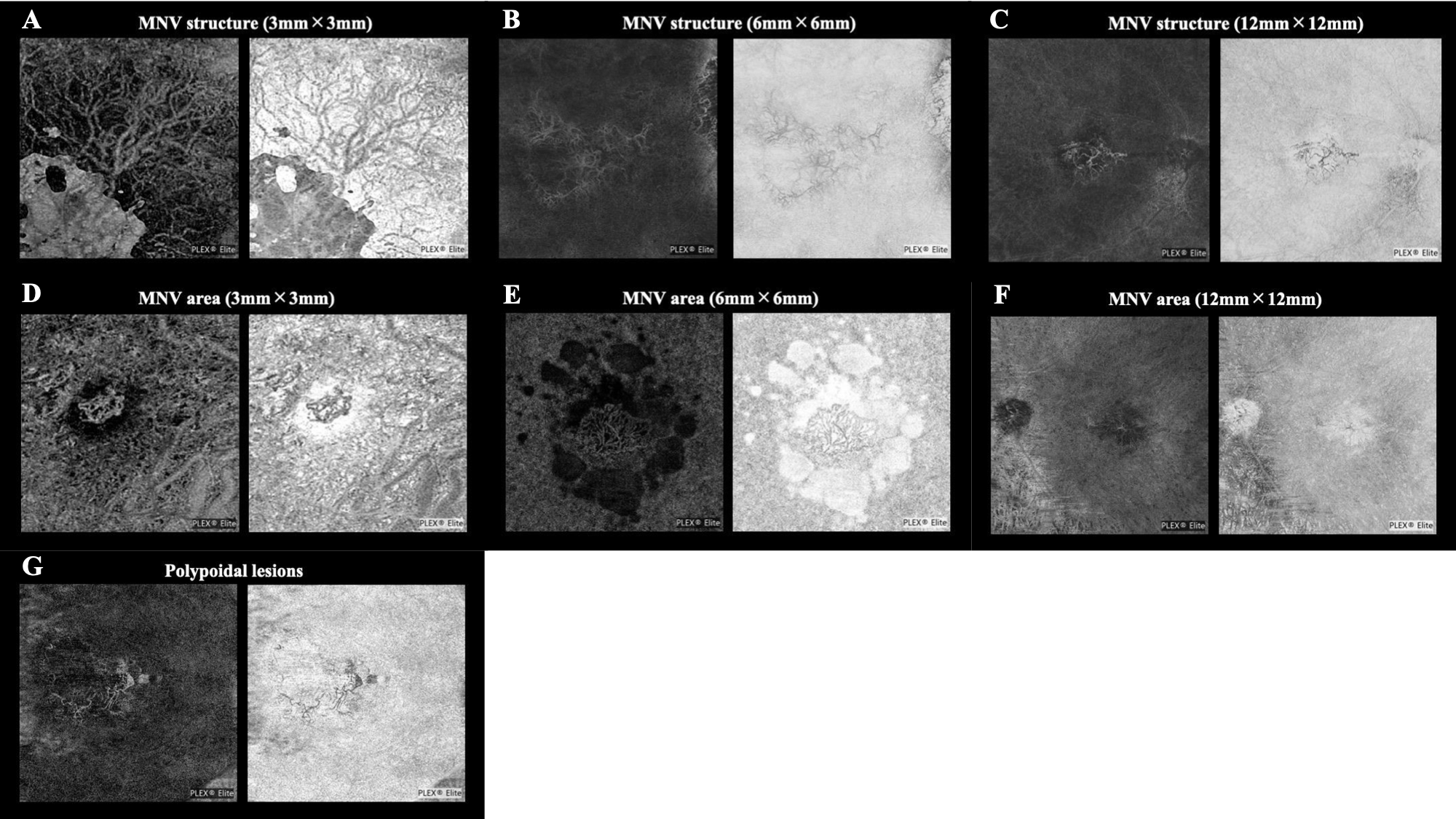

Supplement: Supplementary file 1 [file life-15-01512-s001.zip › suplementary figure.tiff]
